# Supplementary material for: Maternal serum progesterone and BMI are associated with neonatal sex ratios following single frozen embryo transfer
Source: PLoS One. 2026 Apr 9;21(4):e0346711. doi: 10.1371/journal.pone.0346711 (PMC13065069; doi:10.1371/journal.pone.0346711)
Supplement: Attachment — Submitted filename: plos-Czech et al 25.11.09-revised.docx [file pone.0346711.s001.docx]

**Maternal Serum Progesterone and BMI: Independent and Combined Associations with Neonatal Sex After Single Frozen-thawed Embryo Transfer**

**Running title: Progesterone, BMI, and Neonatal Sex after FET**

**Authors: Robert Czech¹, Dariusz Wójcik¹, Tomasz Skweres¹, Wojciech Śliwiński¹, Dorota Zamkowska¹, and Przemysław Ciepiela²***

¹ Gameta Gdynia Center of Health, Świętego Piotra 21 Street, 81-347 Gdynia, Poland
² Bocian Fertility Clinic, Gynecology and Obstetrics, Plac Brama Portowa 1 Street, 70-255 Szczecin, Poland

* Corresponding author:
Przemysław Ciepiela
Bocian Fertility Clinic, Gynecology and Obstetrics
Plac Brama Portowa 1 Street, 70-255 Szczecin, Poland
Email: przemyslaw.ciepiela@gmail.com

Acknowledgements: The authors have reviewed and edited the output and take full responsibility for the content of this publication.

Funding: This research received no external funding

Author Contributions: Conceptualization, R.C. and P.C.; methodology, R.C and P.C..; software, R.C.; validation, R.C. and P.C.; formal analysis, R.C., D.W., T.S, W.Ś., D.Z and P.C.; investigation R.C., D.W., T.S, W.Ś. and D.Z,; resources, R.C.; data curation, R.C.; writing—original draft preparation, P.C.; writing—review and editing, R.C, D.Z. and P.C.; visualization, R.C and P.C; super-vision, D.W.; project administration, R.C and P.C.; funding acquisition, R.C., D.W. and P.C. All authors have read and agreed to the published version of the manuscript.

Conflicts of Interest: The authors declare that they have no conflicts of interest.

**Abstract**

**Background**
The biological mechanisms behind sex ratio variation in assisted reproductive technologies (ART) are not well understood. This study investigated whether maternal serum progesterone (PRG) and body mass index (BMI) are related to neonatal sex after frozen–thawed embryo transfer (FET).

**Methods**
We conducted a retrospective cohort study of 998 single blastocyst FET cycles performed under a freeze-all strategy from 2019 to 2023. Maternal BMI and serum PRG measured on the day of transfer were analyzed in relation to neonatal sex. Receiver operating characteristic (ROC) analyses identified predictive thresholds. Associations were evaluated using multivariable logistic regression models adjusted for potential confounders.

**Results**
ROC analyses identified PRG ≤ 21.11 ng/mL and BMI ≤ 21.30 kg/m² as the optimal cut-offs for predicting male birth. Low PRG was independently associated with a higher odds of male offspring (OR = 1.61; 95% CI: 1.09–2.36; p = 0.016), as was low BMI (OR = 2.33; 95% CI: 1.61–3.40; p < 0.001). When both factors were present, the likelihood of male birth increased further (OR = 2.30; 95% CI: 1.39–3.80; p = 0.001). A high BMI reduced the association between low PRG and male birth, while an elevated PRG weakened the connection between low BMI and neonatal sex. Female births were correspondingly less common under these conditions (OR = 0.43; 95% CI: 0.26–0.72; p = 0.001).

**Conclusions**
Maternal progesterone levels and BMI together influence neonatal sex ratios after FET. These findings imply that endocrine and metabolic environments influence embryo–endometrium interactions in a sex-specific way and open new pathways for research into developmental programming in ART.

**Keywords:** Frozen embryo transfer; Maternal serum progesterone; Body mass index; Sex ratio at birth; Assisted reproductive technology; Embryo–endometrium interaction;

**Introduction**

Assisted reproductive technologies (ART) have made significant progress in recent decades, greatly enhancing outcomes for infertility treatments and broadening our understanding of early human development. Innovations in embryology labs, such as time-lapse embryo monitoring, have transformed in vitro fertilization (IVF) from just a clinical procedure into a valuable tool for studying human reproductive biology [1,2]. Variation in neonatal sex ratios has become an unexpected aspect of assisted reproductive technologies (ART), raising important questions about embryo development and maternal biology [3,4]. Although live birth rates remain the leading indicator of ART success, subtle differences in the secondary sex ratio (SSR), the ratio of male to female live births, have been observed [3–6]. These changes are not only biologically interesting but also clinically significant, as neonatal sex has been associated with perinatal outcomes and long-term health risks [3,5–7].

Several ART-related factors seem to influence SSR. Blastocyst transfer, especially of high-quality embryos, has been linked to a higher rate of male births.While intracytoplasmic sperm injection (ICSI) may reduce this trend or even promote female births, particularly when lower-grade trophectoderm or expansion scores are involved [3,4,6,8]. These observations suggest that embryo handling and developmental dynamics may have sex-specific effects, possibly through epigenetic mechanisms with long-lasting consequences.

Embryonic contributions alone, however, cannot fully account for the observed variation in sex ratios. Male embryos are sometimes reported to reach the blastocyst stage more efficiently but may carry more chromosomal abnormalities [6], while female embryos may show higher rates of aneuploidy [5,9]. Importantly, implantation and ongoing pregnancy rates appear to be largely unaffected by embryonic sex. Since the primary sex ratio (PSR) at fertilization is approximately equal, deviations in SSR are likely influenced during post-fertilization development, implantation, or very early pregnancy [5]. These findings highlight the potential role of maternal physiology in affecting neonatal sex.

Among maternal characteristics, body mass index (BMI) has emerged as a potential factor influencing offspring sex. Our previous research showed that higher maternal BMI was associated with a greater likelihood of female births [10]. This suggests that metabolic factors might influence the intrauterine environment, thereby affecting sex-specific survival or implantation. At the same time, progesterone (PRG) plays a vital role in implantation and endometrial receptivity, and experimental data suggest that sex-specific responses to maternal hormones could influence embryo viability [7,11–14]. However, the combined effects of BMI and progesterone on SSR in ART have not been extensively studied.

In this retrospective cohort study, we investigated the independent and combined effects of maternal BMI and serum progesterone levels on the day of embryo transfer with neonatal sex following frozen-thawed embryo transfer (FET) cycles. To minimize confounding factors, only the first FET cycles were included, using a fully artificial, hormone-controlled endometrial preparation to eliminate the impact of ovarian stimulation. Cycles involving preimplantation genetic testing (PGT) were excluded to preserve natural embryo selection processes. By isolating these variables, our goal was to provide new insights into how maternal metabolic and hormonal environments influence embryo–endometrium interactions and, ultimately, neonatal sex outcomes in ART.

**Materials and Methods**

**Study design and participants**

This retrospective cohort study analyzed 408 singleton live births from 998 consecutive single frozen-thawed embryo transfer cycles conducted between 2019 and 2023 at a tertiary fertility center. All transfers employed a freeze-all strategy, utilizing top-quality embryos selected through standardized morphological grading. Exclusion criteria included: oocyte donation, preimplantation genetic testing (PGT-A/PGT-M), and maternal risk factors for recurrent pregnancy loss, such as genetic abnormalities, antiphospholipid syndrome, uterine synechiae, or Müllerian malformations. Clinical and embryological data were collected from the electronic medical record system (i-IVC database) on February 15, 2025. Before analysis, the dataset was fully anonymized to ensure that investigators could not access any information that would directly identify individual participants, both during and after data collection. Ethical approval was obtained from the Bioethics Committee of the District Medical Chamber in Gdańsk, Poland (Ref. No. KB-43/21).

**Ovarian stimulation, embryo culture, and vitrification**

Controlled ovarian stimulation (COS) used either a GnRH agonist (Gonapeptyl 0.1 mg; Ferring Pharmaceuticals, Germany) or antagonist (Cetrotide 0.25 mg; Merck Serono, Germany) protocol, combined with recombinant FSH (150–225 IU; Gonal-F, Merck Serono, Switzerland; or Puregon, Organon, Netherlands), titrated to baseline anti-Müllerian hormone (AMH) levels. Final oocyte maturation was triggered with recombinant hCG (250 μg; Ovitrelle, Merck Serono, France) or GnRH agonist (0.2 mg Gonapeptyl). All oocytes underwent intracytoplasmic sperm injection.

Embryos were cultured to the blastocyst stage and graded per the Istanbul consensus [15], assessing expansion (Grades 1–6), inner cell mass (ICM: A–C), and trophectoderm (TE: A–C). Senior embryologists documented morphology( revise the sentence). Blastocysts were vitrified using Kitazato VT601 media and warmed with VT602 media per the manufacturer’s protocol, 2–3 h before transfer. Single-embryo transfer was performed regardless of maternal age or reproductive history.

**Endometrial preparation and luteal support**

All FET cycles used a standardized hormone replacement therapy protocol. Estradiol (2 mg, Estrofem; Novo Nordisk Healthcare AG, Switzerland) was administered intravaginally twice daily from cycle day 2. Transvaginal ultrasound on cycle days 13–15 confirmed endometrial thickness.

Patients with a trilaminar endometrium ≥8 mm initiated luteal support with micronized vaginal progesterone (600 mg daily; Luteina, Adamed, Poland) plus oral dydrogesterone (30 mg daily; Duphaston, Solvay Pharmaceuticals, UK). Progesterone supplementation started on the day of serum progesterone testing. It continued through embryo transfer, scheduled on day 5 of progesterone administration (14:00–15:00). Serum progesterone was measured 1–2 h before transfer, maintaining a consistent interval from the morning dose (6:00–7:00). Luteal support was continued until serum β-hCG testing at 12–14 days, and in confirmed pregnancies, up to 10–12 weeks of gestation. Intrauterine pregnancy was confirmed by ultrasound at 8 weeks.

Explain the models group briefly (mentioned in Table-2)

**Study variables and outcomes**

The primary outcome was neonatal sex (male vs. female) at delivery. The main exposures were maternal serum progesterone concentration (ng/mL) on the day of embryo transfer and maternal BMI (kg/m²).

Secondary variables included maternal age, AMH concentration, sperm concentration, and progressive motility (WHO 5th edition, Grade A), endometrial thickness, and embryo quality (Gardner grading).

**Statistical analysis**

Categorical variables were summarized as counts and percentages; continuous variables as medians and interquartile ranges (IQRs). Missing data were excluded. Receiver operating characteristic (ROC) analysis was used to identify optimal cut-off values for progesterone and BMI, using male live birth as the positive outcome Logistic regression models adjusted for confounders estimated associations. Advanced methods were applied to strengthen causal inference:

- Targeted Maximum Likelihood Estimation (TMLE) to assess exposure effects,
- Inverse Probability Weighting (IPW) to reduce selection bias,
- E-value analysis to test sensitivity to unmeasured confounding, and
- Placebo testing to detect residual bias.

All analyses were performed in R Statistical Software (v4.3.3; R Core Team, 2024) under Windows 11 Pro (build 26100). Detailed R code and packages are provided in the Supplementary Materials.

**Results**

**Baseline characteristics**

A total of 408 singleton neonates were analyzed, derived from 998 consecutive frozen-thawed single embryo transfer cycles conducted between 2019 and 2023. Maternal age at FET ranged from 23 to 44 years (median 33.0, IQR 30.0–36.0), while maternal BMI varied from 16.9 to 43.9 kg/m² (median 22.4, IQR 20.5–25.2). Anti-Müllerian hormone levels ranged from 0.24 to 30.0 ng/mL (median 3.63, IQR 2.40–5.57). Semen parameters were variable, with a median sperm concentration of 25.8 million/mL (IQR 7.2–59.6) and progressive motility (Grade A) at 19% (IQR 9.0–30.0). At the start of progesterone treatment, endometrial thickness ranged from 6 to14 mm (median 10, IQR 9–11). Embryo quality was consistently high, with 93.9% expansion Grade 4, 82.8% ICM Grade A, and 80.6% TE Grade A. The neonatal sex ratio was balanced, with 197 males (48.3%) and 211 females (51.7%), providing sufficient power for sex-stratified analyses (Table 1).

**Thresholds for progesterone and BMI**

Receiver operating characteristic analyses identified serum progesterone ≤ 21.11 ng/mL (AUC = 0.56) and BMI ≤ 21.30 kg/m² (AUC = 0.58) as optimal thresholds for predicting male birth. These AUC values reflect limited discriminative ability, and the thresholds are not used for clinical diagnosis. Instead, they serve to define biologically plausible exposure groups for causal modelling.

**Independent associations of progesterone and BMI**

In Model 1, low serum progesterone (≤ 21.11 ng/mL) was significantly linked to male birth (RR = 1.28, 95% CI: 1.04–1.58, p = 0.018; OR = 1.61, 95% CI: 1.09–2.36, p = 0.016). Average treatment effect analyses supported this result (ATT = 0.13, p = 0.009; ATC = 0.10, p = 0.041), and bootstrap confidence intervals confirmed the robustness of the findings. In Model 2, low maternal BMI (≤ 21.30 kg/m²) independently increased the likelihood of male birth (RR = 1.51, p < 0.001; OR = 2.33, 95% CI: 1.61–3.40, p < 0.001). The effect was further supported by strong average treatment estimates (ATT and ATC = 0.21, p < 0.001).

**Concurrent exposure to low progesterone and low BMI**

In Model 3, the simultaneous presence of low PRG and low BMI was linked to the highest likelihood of male birth. The expected probability of a male neonate was 65% with exposure compared to 45% without exposure (ATE = 0.20, p = 0.001), resulting in an OR of 2.30 (95% CI: 1.39–3.80, p = 0.001). These findings were consistent across bootstrap and influence-curve analyses.

**Progesterone–BMI interplay in discordant conditions**

Model 4 assessed low PRG in women with high BMI. No significant association with neonatal sex was found (RR = 0.94, p = 0.621; OR = 0.90, p = 0.619), suggesting that higher BMI may attenuate the impact of low progesterone levels. Model 5 showed high PRG in women with low BMI. There was a non-significant trend toward a higher chance of male births (OR = 1.54, p = 0.113), but the result was not statistically significant.

**Sex-specific inversion**

Analyses from the female offspring perspective demonstrated a complementary pattern. The conditions favouring male births (low PRG and low BMI, Model 3) were associated with a decreased probability of female births (OR = 0.43, 95% CI: 0.26–0.72, p = 0.001). This sex-specific inversion underscores the biological plausibility of the findings.

Visual representations of the model-based odds ratios for male and female births are shown in Figures 1 and 2, respectively.

**Please, specify the Table/Figure when presenting values.**

**Table 1. Baseline characteristics of the study population (n = 408)**

(Values presented as median [IQR] or number [%])

| **Characteristic** | **n** | **Value** |
| --- | --- | --- |
| **PRG 5 days post-FET (ng/mL)** | 408 | 20.40 (15.65–25.38)¹ |
| **Neonate sex:** | 408 |  |
| - Boy |  | 197 (48.28%)² |
| - Girl |  | 211 (51.72%)² |
| **Maternal age at FET (years)** | 408 | 33.00 (30.00–36.00)¹ |
| **Maternal BMI (kg/m²)** | 408 | 22.40 (20.50–25.23)¹ |
| **Maternal AMH (ng/mL)** | 408 | 3.63 (2.40–5.57)¹ |
| **Semen Concentration (million/mL)** | 408 | 25.80 (7.20–59.60)¹ |
| **Semen Motility Grade A (%)** | 408 | 19.00 (9.00–30.00)¹ |
| **Endometrial thickness (mm)** | 408 | 10.00 (9.00–11.00)¹ |
| **Embryo 1 expansion grade:** | 408 |  |
| - Grade 1 |  | 1 (0.25%)² |
| - Grade 2 |  | 1 (0.25%)² |
| - Grade 3 |  | 21 (5.15%)² |
| - Grade 4 |  | 383 (93.87%)² |
| - Grade 5 |  | 2 (0.49%)² |
| **Embryo 1 inner cell mass (Node):** | 408 |  |
| - Grade 1 |  | 338 (82.84%)² |
| - Grade 2 |  | 70 (17.16%)² |
| **Embryo 1 trophoectoderm grade:** | 408 |  |
| - Grade 1 |  | 329 (80.64%)² |
| - Grade 2 |  | 75 (18.38%)² |
| - Grade 3 |  | 4 (0.98%)² |

^1^data are presented as median (IQR), reflecting the 25th and 75th percentiles;
^2^ data are presented as n (%), where n is the number of observations in the category and % is the percentage of the total sample. Abbreviations: PRG = Progesterone; FET = Frozen Embryo Transfer; BMI = Body Mass Index; AMH = Anti-Müllerian Hormone. Embryo Grading: Expansion Grade, Inner Cell Mass, and Trophectoderm Grade are based on standard embryological scoring systems.

**Table 2. Stratified models assessing the risk of male neonatal birth after FET.**

| **Model** | **Definition** | **RR (95% CI)** | **p-value** | **OR (95% CI)** | **p-value** |
| --- | --- | --- | --- | --- | --- |
| **1** | PRG ≤ 21.11 (any BMI) | 1.28 (1.04–1.58) | 0.018 | 1.61 (1.09–2.36) | 0.016 |
| **2** | BMI ≤ 21.30 (any PRG) | 1.51 (1.26–1.81) | <0.001 | 2.33 (1.61–3.40) | <0.001 |
| **3** | PRG ≤ 21.11 and BMI ≤ 21.30 | 1.46 (1.19–1.78) | <0.001 | 2.30 (1.39–3.80) | 0.001 |
| **4** | PRG ≤ 21.11 and BMI > 21.30 | 0.94 (0.77–1.17) | 0.621 | 0.90 (0.60–1.36) | 0.619 |
| **5** | PRG > 21.11 and BMI ≤ 21.30 | 1.23 (0.97–1.57) | 0.088 | 1.54 (0.90–2.61) | 0.113 |

PRG = progesterone; BMI = body mass index; RR = relative risk; OR = odds ratio; CI = confidence interval.

**Figure 1. Odds ratios (OR) for male birth according to maternal serum progesterone and BMI strata (Models 1–5). Error bars represent 95% confidence intervals.**

**Figure 2. Odds ratios (OR) for female birth according to maternal serum progesterone and BMI strata (Models 1–5). Error bars represent 95% confidence intervals.**

**Discussion**

This retrospective cohort study investigated the relationships between maternal serum progesterone levels, body mass index, and neonatal sex following frozen–thawed embryo transfer. Both factors independently affected outcomes, and their combination identified a maternal profile associated with a higher chance of male births. Low PRG and low BMI each independently increased the likelihood of male offspring (Models 1 and 2), and their combined presence further strengthened this association (Model 3). Conversely, high BMI appeared to lessen the impact of low PRG (Model 4), while high PRG in lean women showed only a non-significant trend toward male births (Model 5). Overall, these findings suggest that maternal hormonal and metabolic environments may influence implantation outcomes in a sex-specific manner.

**Progesterone and Endometrial Receptivity**

Progesterone is the primary regulator of endometrial transformation from a proliferative to a receptive state, enabling the establishment of the window of implantation (WOI). In artificial frozen embryo transfer cycles, where corpus luteum–derived hormones are absent, exogenous progesterone alone controls endometrial maturation. Several studies have shown that low serum progesterone levels on the day of transfer are linked to lower implantation and live birth rates [16–18]. Conversely, excessive or untimely progesterone exposure can lead to a premature WOI, disrupting synchrony between the endometrium and the embryo by altering gene expression, cytokine signalling, and the availability of adhesion molecules [19]. Emerging data also indicate that progesterone can cause epigenetic modifications in endometrial cells, potentially affecting receptivity at the molecular level [19].

In our study, higher serum progesterone levels were independently linked to a greater likelihood of female births (aOR = 1.61; 95% CI, 1.09–2.36). This adds a new dimension to the known role of progesterone in implantation by suggesting that the hormonal environment may affect reproductive outcomes differently based on sex. The finding aligns with existing evidence of inherent developmental and metabolic differences between male and female embryos. Previous research has documented sex-related differences in trophoblast invasion, growth rates, timing of implantation, and response to maternal hormonal signals [20–22]. These differences suggest that embryos of different sexes may respond differently to maternal progesterone levels at the time of implantation. Although direct mechanistic data are limited, our results raise the possibility that circulating progesterone concentrations within physiologically relevant ranges for FET could selectively support the implantation of male or female embryos. Further research combining endocrine profiling with molecular analysis of the implantation site is needed to determine whether these early embryo–endometrium interactions are affected by fetal sex.

**Maternal BMI and Neonatal Sex**

Maternal BMI also emerged as an independent factor influencing neonatal sex. Women with a BMI of ≤ 21.30 kg/m² had a significantly higher chance of delivering male offspring (aOR = 2.33; 95% CI, 1.61–3.40), whereas a higher BMI was associated with an increased likelihood of female births. These findings build on our previous observations in a large cohort of single embryo transfers (n = 2679), where we reported an inverse relationship between BMI and the probability of male births in frozen cycles [10]. The current results confirm and refine that association, indicating that maternal metabolic status may have sex-specific effects on embryo implantation or early survival.

Although relatively few studies have examined the relationship between BMI and offspring sex, extensive evidence suggests that BMI is linked to reproductive outcomes in assisted reproduction. Elevated BMI has consistently been associated with reduced implantation and live birth rates [23,24], likely through impaired endometrial receptivity, chronic low-grade inflammation, and altered endocrine signalling. Tarín et al. proposed that the maternal metabolic environment could influence embryo selection or survival in a sex-dependent manner [25], an interpretation consistent with our results. The scarcity of comparable data from natural conception cohorts may reflect the confounding effect of spontaneous early losses, which are less observable in ART populations where implantation events are directly monitored.

These findings collectively reinforce that maternal BMI not only influences overall ART success but may also affect the neonatal sex ratio. The exact mechanisms are still unknown and may involve pathways related to energy balance, inflammatory mediators, and steroid metabolism, which differentially influence male and female embryonic development.

**Progesterone – BMI Interaction and Implantation**

The combined impact of progesterone and BMI proved more informative than either factor alone. The strongest association with male births was observed when low serum progesterone levels coincided with a low maternal BMI, with an adjusted odds ratio of 2.30 (95% CI, 1.39–3.80). This suggests that the simultaneous presence of low progesterone and low BMI may indicate a unique endocrine – metabolic profile that affects the peri-implantation environment in a sex-dependent manner. Although causality cannot be determined, evidence from basic and clinical research supports the biological plausibility of an interaction between progesterone signalling and adiposity.

Experimental studies suggest that progesterone can influence leptin and appetite-related pathways, increasing food intake and fat accumulation [26]. Conversely, a higher BMI may change progesterone pharmacokinetics and tissue exposure by increasing distribution volume, altering liver metabolism, or sequestering in fat tissue [27,28]. Clinical observations report lower circulating progesterone levels in women with obesity undergoing IVF, which could impair optimal endometrial development [28,29]. Beyond blood levels, Mauland et al.[30] found increased progesterone receptor expression in endometrial cancer tissues of women with higher BMI, indicating that adiposity may also affect how the endometrium responds to progesterone . Similar hormonal changes have been seen in men, where obesity is linked to reduced progesterone and disrupted steroid balance [31]. Conversely, Shen et al. demonstrated a positive correlation between BMI and progesterone during pregnancy, showing that this relationship depends on the physiological state and timing of measurement [32]. The study by Bellver et al. [29] found that obese women had significantly lower progesterone concentrations on embryo transfer day compared to non- obese women, possibly linked to hormonal imbalances, including secondary decreases in luteinizing hormone, which may impair progesterone production . Studies by Lim et al.[27] Whynott et al. [28], and Demirel et al. [33] support the association between BMI, progesterone levels, and reproductive outcomes, emphasizing the importance of considering maternal BMI and hormonal status in clinical protocols ..

Together, these studies illustrate a complex, bidirectional relationship between progesterone and adiposity that may extend to the biology of implantation. In our cohort, a higher BMI appeared to weaken the association between progesterone and neonatal sex. In contrast, lean women with high progesterone levels showed only a non-significant trend toward male births. These results suggest that maternal BMI might influence the functional effect of progesterone on implantation success, potentially in a sex-specific way. However, these should be viewed cautiously and considered as hypothesis-generating rather than conclusive.

Finally, the discriminative power of progesterone and BMI for predicting neonatal sex was limited (AUCs 0.56 and 0.58, respectively). This limitation suggests poor diagnostic performance but does not rule out biological significance in multifactorial processes, such as implantation. In our study, ROC-based stratification was used as an exploratory tool to identify biologically plausible subgroups for targeted causal inference, rather than to establish clinical cut-offs.

**Strengths and Limitations**

This study is among the first to examine the combined relationship between maternal serum progesterone, body mass index, and neonatal sex after frozen single embryo transfer. Strengths include a uniform population limited to first FET cycles with single blastocyst transfers and standardized endometrial preparation; sex-blinded embryo selection based solely on morphology; and the use of advanced statistical and causal inference methods, Targeted Maximum Likelihood Estimation, Inverse Probability Weighting, E-values, and placebo testing, to improve robustness and reduce confounding.

Limitations include the retrospective observational design, which precludes causal inference; the restriction to programmed cycles, excluding natural and stimulated cycles with different hormonal dynamics; and a single serum progesterone measurement on the transfer day, thereby limiting understanding of temporal fluctuations and overall exposure. Dydrogesterone, a synthetic progestin used in luteal support, could not be quantified because it is undetectable in standard serum progesterone tests. Although it contributes to overall progestational support, it does not influence serum progesterone levels and was therefore not included in the exposure definition. Serum progesterone is an imperfect proxy for endometrial hormonal status but remains the most practical and clinically accessible marker of luteal function. Finally, ROC-derived AUCs for progesterone and BMI were modest (0.56 and 0.58), indicating limited predictive ability; however, our analyses aimed to examine biologically informed associations rather than clinical prediction, and we identified statistically significant effects within our causal inference framework.

**Conclusions**

In this retrospective analysis of frozen embryo transfer cycles, we found significant links between maternal serum progesterone levels, body mass index, and neonatal sex. A maternal profile with low progesterone and low BMI was associated with a higher chance of male births, while higher progesterone and BMI more often correlated with female births. These results suggest that maternal hormonal and metabolic status during embryo transfer may differentially affect implantation and early development, depending on the sex.

Given the observational nature of this study, causal inference could not be established. Although advanced statistical and causal inference methods were employed to mitigate confounding, the modest predictive performance and absence of dose–response gradients suggest that these findings should be viewed as exploratory and hypothesis-generating. The proposed biological pathways—ranging from progesterone-driven endometrial receptivity and embryo–endometrial synchrony to metabolic signalling and sex-specific implantation dynamics—remain theoretical and need validation through mechanistic and prospective studies.

Importantly, this study neither endorses nor encourages any form of sex selection or intentional modification of maternal physiology to determine fetal sex. It aims to enhance understanding of the natural interactions between maternal endocrine and metabolic factors and embryonic development in assisted reproduction.

Overall, our results emphasize the need for further research into how maternal physiology influences implantation success and secondary sex ratio outcomes in ART. While these findings do not warrant immediate changes in clinical practice, they may inform future studies aimed at refining personalized reproductive care and elucidating the biological factors that influence sex-specific implantation.

**References**

1. Gliozheni O, Hambartsoumian E, Strohmer H, Petrovskaya E, Tishkevich O, de Neubourg D, et al. ART in Europe, 2018: results generated from European registries by ESHRE. Hum Reprod Open [Internet]. 2022;2022. https://doi.org/10.1093/hropen/hoac022

2. Hinojosa SM, Hoces LN, Guzmán L. Time-Lapse Embryo culture: A better understanding of embryo development and clinical application. JBRA Assist Reprod [Internet]. 2021; https://doi.org/10.5935/1518-0557.20210107

3. Cirkel C, König IR, Schultze-Mosgau A, Beck E, Neumann K, Griesinger G. The use of intracytoplasmic sperm injection is associated with a shift in the secondary sex ratio. Reprod Biomed Online. Elsevier Ltd; 2018;37:703–8. https://doi.org/10.1016/j.rbmo.2018.09.009

4. Supramaniam PR, Mittal M, Ohuma EO, Lim LN, McVeigh E, Granne I, et al. Secondary sex ratio in assisted reproduction: an analysis of 1 376 454 treatment cycles performed in the UK. Hum Reprod Open. 2019;2019:1–9. https://doi.org/10.1093/hropen/hoz020

5. Orzack SH, Stubblefield JW, Akmaev VR, Colls P, Munné S, Scholl T, et al. The human sex ratio from conception to birth. Proc Natl Acad Sci U S A. 2015;112:E2102–11. https://doi.org/10.1073/pnas.1416546112

6. Carrasco B, Pons MC, Parriego M, Boada M, García S, Polyzos NP, et al. Male and female blastocysts: any difference other than the sex? Reprod Biomed Online. Elsevier Ltd; 2022; https://doi.org/10.1016/j.rbmo.2022.05.004

7. Supramaniam PR, Mittal M, McVeigh E, Lim LN. The correlation between raised body mass index and assisted reproductive treatment outcomes: A systematic review and meta-analysis of the evidence. Reprod Health. Reproductive Health; 2018;15:1–15. https://doi.org/10.1186/s12978-018-0481-z

8. Zhao J, Shen H, Zhu Q, Liu J, Han J, Yi R, et al. Assisted reproductive technology induces different secondary sex ratio: parental and embryonic impacts. Reprod Health. BioMed Central Ltd; 2023;20. https://doi.org/10.1186/s12978-023-01723-8

9. Luke B, Brown MB, Grainger DA, Baker VL, Ginsburg E, Stern JE. The sex ratio of singleton offspring in assisted-conception pregnancies. Fertil Steril [Internet]. Elsevier Ltd; 2009;92:1579–85. https://doi.org/10.1016/j.fertnstert.2008.08.107

10. Czech R, Wójcik D, Skweres T, Śliwiński W, Ciepiela P. A correlation between the body weight of women undergoing IVF and the gender of offspring. Reprod Biomed Online [Internet]. 2023;103420. https://doi.org/10.1016/j.rbmo.2023.103420

11. Wang M, Liu X, Zhang H, Li L, Liu R, Zhang H, et al. Associated factors of secondary sex ratio of offspring in assisted reproductive technology: a cross-sectional study in Jilin Province, China. BMC Pregnancy Childbirth. BioMed Central Ltd; 2020;20. https://doi.org/10.1186/s12884-020-03373-1

12. Clifton VL. Review: Sex and the Human Placenta: Mediating Differential Strategies of Fetal Growth and Survival. Placenta. 2010;31:S33–9. https://doi.org/10.1016/j.placenta.2009.11.010

13. Goh JY, He S, Allen JC, Malhotra R, Tan TC. Maternal obesity is associated with a low serum progesterone level in early pregnancy. Horm Mol Biol Clin Investig. 2016;27:97–100. https://doi.org/10.1515/hmbci-2015-0030

14. Lou H, Li N, Zhang X, Sun L, Wang X, Hao D, et al. Does the sex ratio of singleton births after frozen single blastocyst transfer differ in relation to blastocyst development? Reproductive Biology and Endocrinology. 2020;18:1–9. https://doi.org/10.1186/s12958-020-00623-x

15. Balaban B, Brison D, Calderón G, Catt J, Conaghan J, Cowan L, et al. The Istanbul consensus workshop on embryo assessment: Proceedings of an expert meeting. Human Reproduction. 2011;26:1270–83. https://doi.org/10.1093/humrep/der037

16. Akaeda S, Kobayashi D, Shioda K, Momoeda M. Relationship between serum progesterone concentrations and pregnancy rates in hormone replacement treatment-frozen embryo transfer using progesterone vaginal tablets. Clin Exp Obstet Gynecol. 2019;46:695–8. https://doi.org/10.12891/ceog4360.2019

17. Boynukalin FK, Gultomruk M, Turgut E, Demir B, Findikli N, Serdarogullari M, et al. Measuring the serum progesterone level on the day of transfer can be an additional tool to maximize ongoing pregnancies in single euploid frozen blastocyst transfers. Reproductive Biology and Endocrinology. Reproductive Biology and Endocrinology; 2019;17:1–8. https://doi.org/10.1186/s12958-019-0549-9

18. Alyasin A, Agha-Hosseini M, Kabirinasab M, Saeidi H, Nashtaei MS. Serum progesterone levels greater than 32.5 ng/ml on the day of embryo transfer are associated with lower live birth rate after artificial endometrial preparation: a prospective study. Reproductive Biology and Endocrinology. Reproductive Biology and Endocrinology; 2021;19:1–9. https://doi.org/10.1186/s12958-021-00703-6

19. Xiong Y, Hu L, Zhang T, Wang M, Xu H, Li TC, et al. Effects of high progesterone in in-vitro fertilization cycle on DNA methylation and gene expression of adhesion molecules on endometrium during implantation window. J Assist Reprod Genet. Springer; 2020;37:33–43. https://doi.org/10.1007/s10815-019-01623-6

20. Sood R, Zehnder JL, Druzin ML, Brown PO. Gene expression patterns in human placenta [Internet]. 2006. http://smd.stanford.edu

21. Baines KJ, West RC. Sex differences in innate and adaptive immunity impact fetal, placental, and maternal health. Biol Reprod. 2023;109:256–70. https://doi.org/10.1093/biolre/ioad072

22. Denis MG, Catteau A, Caillon H, Barrie P. Leptin and its potential interest in assisted reproduction cycles. 2016;22:320–41. https://doi.org/10.1093/humupd/dmv057

23. Maged AM, Fahmy RM, Rashwan H, Mahmood M, Hassan SM, Nabil H, et al. Effect of body mass index on the outcome of IVF cycles among patients with poor ovarian response. International Journal of Gynecology and Obstetrics. John Wiley and Sons Ltd; 2019;144:161–6. https://doi.org/10.1002/ijgo.12706

24. van Duijn L, Rousian M, Hoek J, Willemsen SP, van Marion ES, Laven JSE, et al. Higher preconceptional maternal body mass index is associated with faster early preimplantation embryonic development: the Rotterdam periconception cohort. Reproductive Biology and Endocrinology [Internet]. BioMed Central; 2021;19:1–13. https://doi.org/10.1186/s12958-021-00822-0

25. Tarín JJ, García-pérez MA, Hermenegildo C, Cano A. Tarín JJ, García-Pérez MA, Hermenegildo C, Cano A. Changes in sex ratio from fertilization to birth in assisted-reproductive-treatment cycles. Reprod Biol Endocrinol. 2014;12:56. 2014;1–8.

26. McCrohon JA, Nakhla S, Jessup W, Stanley KK, Celermajer DS. Estrogen and progesterone reduce lipid accumulation in human monocyte- derived macrophages: A sex-specific effect. Circulation. Lippincott Williams and Wilkins; 1999;100:2319–25. https://doi.org/10.1161/01.CIR.100.23.2319

27. Whynott RM, Summers KM, Jakubiak M, Van Voorhis BJ, Mejia RB. The effect of weight and body mass index on serum progesterone values and live birth rate in cryopreserved in vitro fertilization cycles. F S Rep [Internet]. Elsevier Inc.; 2021;2:195–200. https://doi.org/10.1016/j.xfre.2021.02.005

28. Lim MK, Ku CW, Tan TC, Lee YHJ, Allen JC, Tan NS. Characterisation of serum progesterone and progesterone-induced blocking factor (PIBF) levels across trimesters in healthy pregnant women. Sci Rep. Nature Research; 2020;10. https://doi.org/10.1038/s41598-020-59452-y

29. Bellver J, Rodríguez-Varela C, Brandão P, Labarta E. Serum progesterone concentrations are reduced in obese women on the day of embryo transfer. Reprod Biomed Online. Elsevier Ltd; 2022;45:679–87. https://doi.org/10.1016/j.rbmo.2022.05.022

30. Mauland KK, Trovik J, Wik E, Raeder MB, Njølstad TS, Stefansson IM, et al. High BMI is significantly associated with positive progesterone receptor status and clinico-pathological markers for non-aggressive disease in endometrial cancer. Br J Cancer. 2011;104:921–6. https://doi.org/10.1038/bjc.2011.46

31. Martínez-Montoro JI, Molina-Vega M, Asenjo-Plaza M, García-Ruiz MC, Varea-Marineto E, Plaza-Andrade I, et al. Adiposity is associated with decreased serum 17-hydroxyprogesterone levels in non-diabetic obese men aged 18–49: A cross-sectional study. J Clin Med. MDPI; 2020;9:1–12. https://doi.org/10.3390/jcm9123873

32. Shen SW, You JJ, Xu XY, Zhang P, Song C, Shen ZH, et al. Hemodilution and Serum Progesterone Regulation in Males with Obesity. Journal of Inflammation Research. Dove Medical Press Ltd; 2025;18:7235–41. https://doi.org/10.2147/JIR.S525354

33. Demirel C, Özcan P, Tülek F, Timur HT, Pasin Ö. Initiating luteal phase support with sc progesterone based on low serum progesterone on the transfer day in true natural cycle frozen embryo transfers. Front Endocrinol (Lausanne). Frontiers Media SA; 2023;14. https://doi.org/10.3389/fendo.2023.1278042
