# Supplementary material for: Maternal serum progesterone and BMI are associated with neonatal sex ratios following single frozen embryo transfer
Source: PLoS One. 2026 Apr 9;21(4):e0346711. doi: 10.1371/journal.pone.0346711 (PMC13065069; doi:10.1371/journal.pone.0346711)
Supplement: Attachment — Submitted filename: Response to Reviewers .docx [file pone.0346711.s003.docx]

**Dear Academic Editor and Reviewers,**

We sincerely thank the Academic Editor and all three Reviewers for their careful evaluation of our manuscript and their constructive, thoughtful, and detailed comments. We greatly appreciate the recognition of the study’s technical rigor, the appropriateness of the statistical analyses, and the relevance of the research question. In response to the reviewers’ feedback, we have undertaken a thorough revision of the manuscript to improve conceptual clarity, refine interpretive language, strengthen transparency, and explicitly address methodological and biological limitations, while preserving the integrity of the original analyses.

Below, we provide a point-by-point response to each reviewer’s comments. All revisions have been incorporated into the manuscript and are highlighted in the tracked-changes version. Line numbers refer to the Revised Manuscript with Track Changes.

**Response to Reviewer #1**

We thank Reviewer #1 for the thoughtful and detailed critique, particularly regarding biological interpretation, methodological clarity, and the study's framing.

Comment No. 1: The authors present an intriguing premise by correlating neonatal sex with variables measured after embryos have already expressed their genetic sex. While the statistical associations are noteworthy, the biological plausibility requires careful scrutiny. Given that the genetic sex of an embryo is immutable post-fertilization (specifically after the blastocyst stage), it is paramount that the methodology address the specific questions outlined below to clarify the proposed mechanism.

Response No. 1: We thank the Reviewer for this important conceptual comment and fully agree that genetic sex (XX/XY) is determined at fertilization and remains immutable thereafter. Importantly, our study does not propose that maternal serum progesterone levels or BMI determine embryonic sex. Rather, it examines whether variation in the maternal hormonal and metabolic environments during the peri-implantation period is associated with differences in the secondary sex ratio (SSR) observed among liveborn infants.

Our premise is that although genetic sex is fixed at fertilization, the sex ratio at birth may be modified by sex-specific differences in implantation success and/or early pregnancy survival that occur after fertilization. Progesterone plays a central role in endometrial receptivity, decidualization, immune modulation, and early placentation. Although these processes are not inherently sex-specific, accumulating evidence indicates that male and female embryos and placentas differ in early growth dynamics and stress responses, potentially leading to sex-differential vulnerability under suboptimal peri-implantation conditions. Accordingly, any observed association would reflect post-fertilization selection or survival rather than sex determination. To prevent misinterpretation, we have revised the manuscript to explicitly distinguish sex determination from variation in the secondary sex ratio and to consistently use non-causal, associative language throughout the text.

Action taken

Title revision: In response to concerns about conceptual clarity and potential overinterpretation, we revised the title to explicitly emphasize (i) the observational nature of the analysis (“associated with”), (ii) the population-level outcome (“neonatal sex ratios”), and (iii) the post-transfer timeframe.

Revised title: Maternal Serum Progesterone and BMI Are Associated with Neonatal Sex Ratios Following Single Frozen Embryo Transfer

Conceptual clarification in the Discussion (Lines 264–270):

“By isolating these variables within a standardized frozen embryo transfer setting, our aim was to generate hypotheses about how maternal metabolic and hormonal environments may relate to embryo–endometrium interactions and subsequent variation in the secondary sex ratio, rather than to imply the determination of genetic sex.”

Language refinement (Line 270):

Replaced causal phrasing (“affected outcomes”) with associative terminology (“were associated with neonatal sex outcomes”).

Explicit statement in the Conclusions (Lines 408–411):

Added clarification that the study does not support or enable sex selection and that the neonatal sex ratio was evaluated solely as a biological outcome reflecting early developmental and implantation-related processes.

**Comment No. 2:** Introduction. The first two paragraphs of the Introduction should be condensed. They currently discuss factors influencing sex determination before blastocyst formation, which differs from the post-conceptional focus and hypothesis of the present study.
The explanatory content in the final paragraph of the Introduction seems premature; I suggest moving these interpretations to the Discussion section to maintain a clear narrative flow.

**Response No. 2:** We agree with the Reviewer’s suggestion. The Introduction has been condensed to focus more directly on the study rationale, specifically the secondary sex ratio, implantation, and early pregnancy processes, while broader interpretive content has been moved to the Discussion. This restructuring improves narrative focus and ensures a clearer separation between background information and interpretation of findings.

Action taken: The first two paragraphs of the Introduction were shortened to reduce redundancy and limit interpretive statements. One interpretive sentence was moved from the final paragraph of the Introduction to the Discussion, where interpretation of the findings is more appropriate.

Relocated sentence (now Lines 264–268, Discussion):

“By isolating these variables within a standardized frozen embryo transfer setting, our aim was to generate hypotheses about how maternal metabolic and hormonal environments may relate to embryo–endometrium interactions and subsequent variation in the secondary sex ratio, rather than to imply determination of genetic sex.”

**Comment No. 3:** Methodology. The methodology lacks data on BMI and progesterone measurements at the specific time of In Vitro Fertilization (IVF). Including these data points is essential to capture the variables present at the precise moment of genetic sex establishment.

**Response No. 3:** We thank the Reviewer for this important comment and for highlighting the need to clarify the biological rationale for our hypothesis. We fully agree that genetic sex determination occurs at fertilization and remains immutable thereafter, and we emphasize that our study does not propose that maternal progesterone levels or BMI influence genetic sex determination. Instead, our hypothesis centers on a post-zygotic, implantation-stage selection mechanism. Specifically, we propose that maternal hormonal and metabolic conditions at the time of embryo transfer and implantation may be associated with sex-specific differences in implantation success and/or very early survival of embryos whose genetic sex has already been established. Under this framework, any observed differences in neonatal sex ratios reflect differential implantation or survival probabilities of male versus female embryos, rather than an alteration of genetic sex.

We acknowledge that this conceptual distinction may not have been sufficiently explicit in the original manuscript. We have therefore revised the Introduction and Methods to explicitly state that the biologically relevant exposure window in our analysis is the peri-implantation period, rather than fertilization or the establishment of genetic sex. We believe these revisions clarify the biological plausibility of our hypothesis while remaining fully consistent with established embryological principles.

Action taken:

Introduction (Lines 82–85):

Clarified that deviations in the secondary sex ratio are most plausibly influenced during post-fertilization development, implantation, or very early pregnancy rather than at fertilization.

Methods (Lines 107–112):

Explicitly explained why progesterone exposure was defined at the time of embryo transfer and why fertilization-stage measurements were not considered biologically informative for the study objective.

Discussion (Lines 289–292):

Clarified the biological relevance of progesterone concentrations measured around embryo transfer in relation to embryo–endometrium interactions and early survival processes.

**Comment No. 4:** The criteria for selecting the “first transferred embryo” are not clearly defined. Furthermore, the manuscript does not explain why subsequent embryo transfers were excluded from the report. According to the hypothesis presented, subsequent transfers should theoretically yield similar results; omitting them requires justification.

**Response No. 4:** We thank the Reviewer for this important methodological observation. Only the first frozen embryo transfer (FET) per patient was included to ensure statistical and biological independence of observations. Including multiple transfers from the same patient would introduce within-patient correlation and potential cumulative exposure and embryo selection biases, which could distort associations with the neonatal sex ratio.

Although subsequent transfers occur within the same individual, they are not biologically or analytically equivalent to the first transfer. Embryo hierarchy effects (e.g., preferential transfer of higher-quality embryos earlier), changes in endometrial conditions across cycles, and prior pregnancy outcomes may influence implantation dynamics and survival probabilities. Consequently, treating subsequent transfers as independent observations would violate core assumptions of standard regression models and could bias effect estimates.

We therefore restricted the analysis to the first FET per patient as a conservative design choice to maximize interpretability and methodological rigor. This approach is consistent with common practice in reproductive epidemiology when the outcome of interest is assessed at the patient level.

Action taken: Methods (Lines 113–115):

Added an explicit justification for including only the first frozen embryo transfer per patient:

“Only the first frozen embryo transfer per patient was included to avoid within-patient correlation, cumulative exposure bias, and embryo selection effects associated with multiple transfers.”

**Comment No. 5:** Male factor variables at the time of fertilization are not established in the current methodology. These must be included to rule out potential confounding factors that could influence sex ratios.

**Response No. 5:** We thank the Reviewer for raising this point. Male factor variables were considered in the analysis and included as covariates where available, specifically sperm concentration and motility. These parameters were included to account for potential male-related influences on reproductive outcomes. Importantly, existing evidence suggests that male factor infertility has a limited impact on the secondary sex ratio at live birth, particularly in assisted reproductive technology settings, especially in ICSI cycles, where sperm selection and fertilization procedures largely standardize fertilization conditions. As our study focuses on neonatal sex ratio rather than fertilization outcomes, residual male-related effects are expected to be minimal. Nonetheless, we acknowledge that unmeasured male factors cannot be entirely excluded. This limitation is now explicitly addressed in the manuscript to ensure transparency and appropriate interpretation of the findings.

Action taken: Methods (Lines 157–159):

Clarified the inclusion of male factor parameters (sperm concentration and motility) as covariates in the statistical models.

Limitations (Lines 382–385):

Acknowledged the possibility of residual confounding by unmeasured male factors while noting the limited expected impact on secondary sex ratio at live birth.

**Comment No. 6:** Results. Please explicitly state the absolute number (n) of male and female neonates within the “low” and “high” groups for the proposed Models 1 through 3. It is crucial to assess whether a small subset of patients with both low progesterone and low BMI might be disproportionately skewing the overall results of the models.

**Response No. 6:** We agree with the Reviewer that reporting absolute numbers is important for assessing the robustness of the findings. We have therefore added the absolute counts (n) of male and female neonates across the primary exposure strata for Models 1–3. These data show that the observed associations are not driven by a small subgroup of patients, including those with concomitant low progesterone and low BMI.

**Action taken**

Results section (Models 1–3): Added explicit reporting of the absolute numbers of male and female live births within the “low” and “high” exposure groups.

Table 2: Updated to include absolute counts (n) for male and female neonates for each exposure category.

**Comment No. 7:** The implantation rates for both the low and high groups must be reported. This data is critical to support or refute the authors’ hypotheses regarding differential survival or implantation based on sex.

**Response No. 7:** We agree that implantation and early survival are central to the biological interpretation of secondary sex ratio variation. However, because the present analysis was restricted to singleton live births, implantation failure and very early pregnancy loss were not directly observable in this dataset. Consequently, sex-specific implantation rates could not be calculated. As clarified in the manuscript, the neonatal sex ratio at birth was used as a proxy outcome reflecting the cumulative result of sex-specific implantation success and/or very early pregnancy survival. While this approach does not allow direct assessment of implantation rates, it is consistent with prior epidemiological studies examining secondary sex ratio using live-birth data. We now explicitly acknowledge this limitation and emphasize that the findings should be interpreted accordingly.

Action taken:

Introduction (Lines 81–84):

Clarified that implantation failure and very early pregnancy loss could not be directly assessed due to the study design, and justified the use of neonatal sex ratio as a proxy for sex-specific survival during implantation and early gestation.

Discussion:

Reiterated this limitation to ensure appropriate interpretation of the findings and to avoid overextension of mechanistic conclusions.

**Comment No. 8:** Please clarify whether there is any patient overlap between the current study and the self-referenced article cited in the discussion. Potential data duplication should be transparently addressed.

**Response No. 8:** We thank the Reviewer for raising this important point about transparency and potential data overlap. The database used in the present study is substantially larger than that used in our previously published work. Although some patients may appear in both datasets, the current analysis is not a duplication of prior results. Specifically, the present study includes a larger, updated cohort; a restriction to a more homogeneous, methodologically cleaner subgroup, namely patients undergoing their first frozen embryo transfer after a freeze-all strategy; and the incorporation of a new exposure variable, maternal serum progesterone concentration, which was not examined in the previous study. The analytical focus, study question, and primary exposures therefore differ meaningfully from the earlier report. Accordingly, the current work represents a distinct and independent analysis, designed to address a separate biological hypothesis, while benefiting from a refined study population that minimizes within-patient correlation and cumulative transfer bias.

Action taken: In the Discussion section, we added an explicit statement clarifying the relationship between the current study and the previously published work, emphasizing cohort expansion, refined inclusion criteria (first FET only), and the introduction of progesterone as a novel exposure variable.

Lines: 314 – 317: Although the dataset partially overlaps with a previously published cohort, the present study is an independent analysis of an expanded population limited to first frozen embryo transfer cycles and includes maternal serum progesterone concentration as a novel exposure. This design enhances cohort homogeneity and reduces cumulative transfer and embryo selection bias.

**Comment No. 9:** Concluding Observation The authors posit a theory of lower implantation rates for female embryos in the presence of low BMI and low progesterone. Based on this premise, the most robust model to validate this hypothesis would be to determine and compare the implantation rates of embryos with previously known sex (e.g., via PGT-A) against the BMI and Progesterone variables. I recommend addressing why this approach was not taken or how the current model serves as a sufficient proxy.

**Response No. 9:** We agree with the Reviewer that studies using embryos with previously known sex (e.g., via PGT-A) can address complementary and highly informative questions. However, these designs examine a fundamentally different biological context. PGT-A involves embryo biopsy and selection, which alter implantation dynamics and may obscure or override subtle sex-specific selection processes that occur under unmanipulated conditions. The present study was intentionally restricted to non-PGT frozen embryo transfer cycles to preserve natural implantation dynamics, allowing sex-specific differences in implantation success or early survival, if present, to manifest without prior selection. Accordingly, our analysis is framed as hypothesis-generating, using neonatal sex ratio at live birth as a proxy outcome reflecting cumulative post-fertilization selection during implantation and early gestation.

We have clarified this distinction in the Methods and Discussion, and we explicitly acknowledge that PGT-A–based designs are an important direction for future confirmatory research rather than a substitute for the current analytical framework.

Action taken

Methods (Lines 117–120): Clarified the exclusion of PGT cycles and the rationale for preserving unmanipulated implantation conditions.

Discussion: Explicitly discussed the complementary nature of PGT-A–based studies and explained why the neonatal sex ratio at live birth is an appropriate proxy in the present design.

(Lines 365–373): Although implantation rates of embryos with known sex (e.g., following PGT-A) could provide direct evidence of sex-specific implantation differences, such designs involve embryo biopsy and selection, which may substantially alter implantation dynamics and mask spontaneous selection processes. The present study, therefore, focused on non-PGT frozen embryo transfer cycles to preserve unmanipulated embryo–endometrium interactions. In this context, the neonatal sex ratio at live birth serves as a pragmatic proxy outcome reflecting the cumulative result of sex-specific implantation success and very early pregnancy survival. Accordingly, our findings should be viewed as hypothesis-generating and complementary to future PGT-A–based studies designed to directly test sex-specific implantation probabilities

**Response to Reviewer #2**

We thank Reviewer #2 for the positive and thoughtful evaluation of our manuscript and for highlighting its relevance to ongoing discussions about maternal hormonal and metabolic influences in assisted reproductive technology. We also appreciate the careful review of the manuscript and the constructive suggestions to improve clarity and presentation.

**Comment No. 1**: It is a well-designed, informative, and relevant research paper that has sparked new discussions about the impact of maternal hormones and body composition on assisted reproductive technology (ART). Check the manuscript carefully as some corrections are made in the text. Groups need to be explained in detail.

**Response No. 1:** We thank the Reviewer for these encouraging comments and the suggestion to improve clarity in the group definitions. We agree that a more detailed explanation of the exposure groups and analytical models enhances readability and interpretability. The manuscript has been revised accordingly.

Action taken

Methods (Lines 171–175):

Expanded the description of exposure group definitions and provided a clearer explanation of Models 1–5, including their rationale and exploratory nature.

**Comment No. 2:** Table and graph numbers must be indicated while presenting the values in the Results section.

**Response No. 2:** We agree with the Reviewer and appreciate this suggestion to improve clarity and readability of the Results section.

Action taken

Results section: All numerical results now explicitly reference the corresponding tables and figures, including Table 1, Table 2, Figure 1, and Figure 2, wherever values are presented in the text.

**Comment No. 3:** The diagnostic limitations should be made more explicit, especially the interpretation of the low AUC in the ROC and the caution in clinical application.

**Response No. 3:** We agree with the Reviewer and appreciate this important point. The ROC analyses were intended solely for exploratory stratification rather than diagnostic prediction, and the observed AUC values indicate modest discriminatory ability. We have revised the manuscript to more explicitly emphasize these limitations and to caution against clinical interpretation or application of the ROC-derived thresholds.

This is clarified in:

Discussion (Lines 403 – 409): Expanded to explicitly address the modest AUC values, clarify their exploratory nature, and state that ROC-based stratification does not support diagnostic or predictive use.

Conclusions (Lines 416 – 421): Revised to reinforce that the findings are hypothesis-generating and should not be interpreted as clinically actionable or diagnostic.

**Comment No. 4:** The ethical discussion section could be more comprehensively included, since sex determination is a sensitive social issue.

**Response No. 4:** We agree with the Reviewer and appreciate the emphasis on the ethical sensitivity of research involving sex-related outcomes. We have revised the manuscript to more explicitly state that the present study neither supports, enables, nor advocates sex selection, and that the neonatal sex ratio is analyzed solely as a biological outcome reflecting early developmental processes, with no clinical or social application.

Discussion (Lines 407 – 410): Expanded to explicitly address the ethical context of studying neonatal sex ratios and to clarify that the findings should not be interpreted as supporting sex selection or sex determination.

Conclusions (Lines 428 – 431): Added a clear statement reinforcing that the study has no implications for clinical sex selection and should be interpreted exclusively within a biological and epidemiological framework.

**Comment No. 5:** Suggestions for future research could be presented more clearly, such as a prospective cohort design and long-term outcome tracking.

Response No.5 We agree with the Reviewer and appreciate this constructive suggestion. The Discussion has been strengthened to more clearly outline future research directions, including prospective cohort designs, mechanistic studies, and long-term outcome tracking, to further investigate the biological pathways underlying the observed associations.

We proposed directions for future research in the text in lines

Discussion (Lines 421 – 427):

Expanded to explicitly describe future research directions, including prospective cohort studies and investigations of implantation and early survival mechanisms.

Discussion (Lines 432 – 435):

Added reference to the importance of long-term follow-up and outcome tracking to contextualize neonatal sex ratio findings within broader reproductive and developmental frameworks.

**Response to Reviewer #3**

We thank Reviewer #3 for the careful and critical evaluation of our manuscript and for emphasizing the importance of appropriate interpretation when examining sensitive and complex outcomes such as neonatal sex ratio. We appreciate the opportunity to clarify and strengthen the manuscript accordingly.

**Comment No. 1:** Throughout the whole manuscript the authors use language implying a causal relationship (e.g. “influence”, “affect”, “determine”) between serum progesterone/BMI and neonatal sex. Especially given the retrospective character of the study, such wording should be considered as overstatement and is not justified. Having used advanced statistical methodology (TMLE, IPW, E-values), still, it does not establish biological causality—particularly for an endpoint such as neonatal sex, reflecting a series of events (implantation, placentation, miscarriage, fetal development). Additionally, no direct data are provided to support the described sex-specific effects of progesterone at implantation. It is suggested to replace causal language by associative terminology and to present the findings as hypothesis-generating.

**Response No. 1:** We fully agree with the Reviewer’s assessment. Although advanced statistical approaches (including TMLE, IPW, and E-values) were used to strengthen causal inference, these methods do not establish biological causality, particularly for a complex endpoint such as the neonatal sex ratio, which reflects the cumulative outcome of multiple post-fertilization processes, including implantation, placentation, and early pregnancy survival. In line with this, we have systematically revised the manuscript to remove causal terminology (e.g., “influence,” “affect,” “determine”) and replace it with associative language throughout. The findings are now consistently framed as exploratory and hypothesis-generating, and we explicitly acknowledge that the current study design does not provide direct mechanistic or sex-specific data on implantation. These revisions ensure that the interpretation of the results remains appropriately cautious and fully aligned with the study's retrospective observational design.

Action taken: Global revision of terminology: Causal wording was replaced with associative language throughout the Abstract, Results, Discussion, and Conclusions.

Interpretative reframing: The study is now consistently presented as hypothesis-generating, with explicit acknowledgment of the limitations in inferring biological mechanisms or sex-specific effects at implantation.

**Comment No. 2:** Additionally, authors state that the endometrial preparation comprised of vaginal estradiol administered twice daily, and for luteal support, vaginal micronised progesterone and oral dydrogesterone were used. Please specify the dosing of estradiol in the studied population and explain the rather untypical vaginal route of its administration.

**Response No. 2:** We thank the Reviewer for this comment and agree that clearer reporting of estradiol dosing and administration is important for methodological transparency. We have clarified both the dose and the rationale for the vaginal administration of estradiol in the study population.

Action taken: Methods (Lines 140–142):

Specified that estradiol was administered at a dose of 2 mg intravaginally twice daily and added a brief rationale for this route of administration, reflecting local clinical practice aimed at achieving effective endometrial exposure while minimizing systemic effects.

**Comment No. 3:** As per using oral dydrogesterone in the luteal support, it is worth noting that both the medication itself as well as its active metabolite are potent gestagens. Lack of cross-reactivity in progesterone assays does not imply lack of their biological effect. Neumann et al. (Human Reproduction, 2022) demonstrated an inverse association between BMI and DHD concentrations, indicating that body composition and metabolism may modulate dydrogesterone exposure. Therefore, it cannot be excluded that part of the observed association between BMI, progesterone, and neonatal sex may be influenced by unmeasured variability in dydrogesterone/DHD exposure and it should be explicitly stated within the text. It should be also included within the limitations section naming dydrogesterone as a potential confounder / effect modifier.

**Response No. 3:** We fully agree with the Reviewer and thank them for highlighting this important pharmacological consideration. Although dydrogesterone and its active metabolite (DHD) do not cross-react with serum progesterone immunoassays, they exert biologically relevant progestational effects at the endometrial level. As the Reviewer noted, emerging evidence suggests that BMI-related metabolic variability may influence dydrogesterone/DHD exposure, which could theoretically modify implantation dynamics or early pregnancy processes.

Accordingly, we explicitly acknowledge that variability in dydrogesterone exposure is a potential unmeasured confounder or effect modifier of the observed associations among maternal BMI, measured serum progesterone, and neonatal sex ratio. This limitation is now clearly stated in the manuscript to ensure appropriate interpretation of the findings.

Action taken in Limitations (Lines 397 – 403):

Added an explicit discussion of oral dydrogesterone as a potential confounder and effect modifier, including reference to BMI-related metabolic variability and its possible influence on progestational exposure independent of measured serum progesterone.

**Comment No 4:** Specific rewording was proposed.

**Response No. 4:** We adopted the reviewer’s suggested wording or closely equivalent phrasing to ensure appropriate caution and clarity.

We believe these revisions substantially strengthen the manuscript by clarifying its conceptual framework, appropriately limiting causal interpretation, and transparently addressing methodological constraints. We are grateful to the Academic Editor and Reviewers for their insightful feedback, which has significantly improved the quality of this work. We hope the revised manuscript now meets the criteria for publication in PLOS ONE.

Sincerely,

Przemysław Ciepiela, MD, PhD

on behalf of all authors
