# Supplementary material for: Maternal serum progesterone and BMI are associated with neonatal sex ratios following single frozen embryo transfer
Source: PLoS One. 2026 Apr 9;21(4):e0346711. doi: 10.1371/journal.pone.0346711 (PMC13065069; doi:10.1371/journal.pone.0346711)
Supplement: Attachment — Submitted filename: Response to Reviewers.docx [file pone.0346711.s004.docx]

Dear Editor and Reviewers,

We would like to express our sincere gratitude for the time, expertise, and thoughtful consideration you devoted to reviewing our manuscript. Your insightful comments have been invaluable in improving the clarity, precision, and scientific rigor of our work. We truly appreciate the opportunity to revise the manuscript and are grateful for your constructive guidance, which has significantly strengthened the final version.

We believe that, as a result of your recommendations, the manuscript now presents a clearer and more methodologically rigorous account of our findings. In our view, the study provides novel, clinically relevant evidence in assisted reproductive technology, particularly regarding the association among maternal progesterone levels, BMI, and neonatal sex outcomes after frozen embryo transfer. We hope that the revised version better reflects both the scientific value and the associative nature of our findings. Below, we summarize the key revisions made in response to the remaining comments:

**Response to Reviewer #2**

As suggested, we revised the sentence in the Methods section to improve clarity and scientific precision. The original wording (Line 127):

“Senior embryologists documented morphology.”

has been replaced with:

“Senior embryologists prospectively recorded blastocyst morphological parameters in the laboratory database.”

**Response to Reviewer #3**

We sincerely thank Reviewer #3 for the careful evaluation and for highlighting the importance of avoiding causal overstatements. We have implemented the suggested edits verbatim to ensure that the language throughout the Discussion strictly reflects the associative nature of the findings. The following revisions were made exactly as recommended:

Line 247 revised to:

“Both factors were associated with neonatal sex outcomes, and their joint presence was associated with higher odds of male births.”

Line 248 revised to:

“Low PRG and low BMI were each independently associated with higher odds of male offspring (Models 1 and 2); when both were present, the odds were highest (Model 3).”

Line 387 revised to:

“Male births were more frequently observed among pregnancies with low progesterone and low BMI, while female births were more frequently observed among pregnancies with higher progesterone and higher BMI.”

In addition, we carefully reviewed the entire manuscript to ensure consistent use of associative rather than causal language. Finally, in accordance with the Editor’s instructions, we have included the following files with our resubmission:

A detailed point-by-point response to all comments raised by the Academic Editor and Reviewers, uploaded as a separate file labeled “Response to Reviewers.” A marked-up version of the manuscript highlighting all revisions, uploaded as “Revised Manuscript with Track Changes.” A clean, unmarked version of the revised manuscript, uploaded as “Manuscript.”

We are grateful for the opportunity to revise and resubmit our work and thank you again for your constructive feedback and consideration. We look forward to your decision.

Sincerely,

Przemysław Ciepiela, MD, PhD

On behalf of all authors
